# Piperine as a neuroprotective functional component in rats with cerebral ischemic injury

**DOI:** 10.1002/fsn3.1185

**Published:** 2019-09-10

**Authors:** Shiyao Hua, Jiayue Liu, Yiwei Zhang, Juan Li, Xinhui Zhang, Lin Dong, Yunsheng Zhao, Xueyan Fu

**Affiliations:** ^1^ School of Pharmacy Ningxia Medical University Yinchuan China; ^2^ School of Basic Medical Sciences Ningxia Medical University Yinchuan China; ^3^ Ningxia Engineering and Technology Research Center for Modernization of Hui Medicine Yinchuan China; ^4^ Key Laboratory of Hui Ethnic Medicine Modernization Ministry of Education (Ningxia Medical University) Yinchuan China

**Keywords:** apoptosis, neuroprotective effects, permanent middle cerebral artery occlusion, piperine

## Abstract

Long pepper (*Piper longum* L.) and black pepper (*Piper nigrum* L.) plants are commonly used as spices around the world and have also been postulated to have medicinal effects. Piperine, as the major alkaloid of *P. nigrum* and *P. longum*, has gained wide attention of the medical community and culinary enthusiasts. This study seeks to determine the effects of piperine on neuronal apoptosis in peri‐infarcted cerebral cortices of rats with permanent middle cerebral artery occlusion (pMCAO) injury. Evaluation of the different behavioral components was conducted after pMCAO. 2, 3, 5‐Triphenyltetrazolium chloride (TTC) was used to evaluate the area of cortical ischemia. Gross histopathological changes, as well as microscopic neuronal changes, were observed in brain tissue samples. The protein expression of Caspase‐3, Caspase‐9, Bax, Bcl‐2, and Cytochrome C (Cyt‐c) was analyzed using western blotting. The findings reveal that rats that received piperine treatment show markedly decreased neurological deficit, less ischemia‐induced cellular damage, as well as smaller areas of cerebral infarction, with less severe macro and microcellular cerebral structural changes. Western blotting analysis reveals that piperine administration inhibits Bax, while enhancing Bcl‐2 expression. The protein expression of Caspase‐3, Caspase‐9, and Cyt‐c was also found to be significantly inhibited. We conclude that piperine may provide several beneficial neuroprotective effects that warrant further investigation.

## 1 INTRODUCTION

Ischemic cerebral injury resulting from cardiac arrest or stroke remains a significant cause of human morbidity, as a result of its debilitating and often permanent cognitive and motor damage (Chu et al., 2016; Shi, Yang, Chen, Chen, & Tu, 2014). Neuronal apoptosis is a key feature of cerebral ischemia, a phenomenon that involves both the innate and adaptive immune systems (Costantino & Josef, 2012). Strokes are a large burden on socioeconomic and healthcare systems, as they are the leading cause of morbidity and mortality in China and the second leading cause globally (GBD, 2016 Causes of Death Collaborators 2017; GBD, 2016 DALYs, & HALE Collaborators 2017). Therefore, the discovery of novel therapeutic interventions that may improve stroke outcomes is of critical importance.

Apoptosis exerts a large influence on ischemic stroke pathogenic mechanism. Therefore, protecting neurons from apoptosis could be a reasonable remedial goal (Broughton, Reutens, & Sobey, 2009). Among the two general apoptosis pathways, the mitochondrial‐mediated apoptosis pathway plays a crucial role in neuronal cell apoptosis after ischemic stroke (Graham & Chen, 2001). Caspase‐9 and Caspase‐3 are activated through the release of mitochondrial Cyt‐c, which ultimately results in apoptosis progression (Reed, 2000). Moreover, Bcl‐2 family proteins can control apoptosis by adjusting Cyt‐c release, as well as through mitochondrial permeability (Hyo‐Soon et al., 2008). Once the death signal is received, Bax, the proapoptotic protein, accelerates Cyt‐c release from mitochondria into the cytosol. However, Bcl‐xl, the antiapoptotic protein, prevents this release (Zhang et al., 2010).

Pepper is an ubiquitous spice that is used worldwide. In countries such as India and China, pepper is also considered as an important medicinal ingredient (Mao, Huang, Zhong, Xian, & Ip, 2014; Rameshkumar, Anuaravind, & Mathew, 2011). Piperine (1‐piperoyl piperidine) is a nitrogen‐containing alkaloid, derived from fruits of the long pepper (*Piper longum*), black pepper (*Piper nigrum*), and other *Piper* species (family: Piperaceae). The piperine content in the whole pepper berry reportedly ranges from 2% to up to 9%, depending on the variety. Piperine has historically been a component of the human diet, and is responsible for the characteristic pungent sensory effect of pepper. Piperine has been documented to have several pharmacological functions, such as antidepressive (Li et al., 2007), antiepileptic (Liu et al., 1986), anti‐inflammatory (Bae et al., 2010), anti‐oxidant (Zhang et al., 2017), and anticonvulsant (Mishra, Punia, Bladen, Zamponi, & Goel, 2015) properties.

Based on the many effects of piperine on the nervous system mentioned, its neuroprotective effect has also been studied. This study seeks to further explore the neuroprotective effect of piperine in pMCAO animal models, a phenomenon that has yet to be reported in detail.

## 2 MATERIALS AND METHODS

### 2.1 Animals

Experimental protocols were reviewed by the Ethics Committee of Ningxia Medical University, Ningxia (Ethics approval, 2015‐156). Specific pathogen‐free Sprague‐Dawley rats (260–310 g; license no. SCXK (NING) 2012‐0001) were bought from the Experimental Animal Center of Ningxia Medical University. The rats were reared in standard laboratory cages under 12‐hr light–dark cycles with free flow of water and food, at consistent temperatures (22 ± 1°C) and moderate ambient humidity (55% ± 5%).

### 2.2 Chemicals and drugs

Piperine (≥98% purity) was procured from the Shanghai De Bai Chemical Technology Co., Ltd. Nimodipine tablets were purchased from Bayer, Germany. TTC was bought from Solarbio Life Sciences (Beijing Solarbio Science & Technology Co., Ltd). BCA protein quantitative kits and total protein extraction kits were purchased from Ken Gen Biotech. Co. Ltd. Primary antibodies of Caspase‐3, Caspase‐9, Bax, Bcl‐2, Cyt‐c, and β‐actin, as well as the secondary antibody of horseradish peroxidase‐conjugated goat antirabbit IgG were obtained from Proteintech Group, Inc. SuperSignal West Pico was bought from Thermo Scientific, US.

### 2.3 Construction of a pMCAO model

The pMCAO modeling on methods widely described in existing literature was slightly modified (Luo et al., 2014). The rats were anesthetized using intraperitoneal injections of chloral hydrate (7%, 0.5 ml/100 g) after 12 hr of abstinence from food (free access to fluids allowed). Prior to incision, the rats were placed in a supine position. A midline incision was made over the skin of the neck, which allowed for the identification and preservation of the right common, internal (ICA) and external carotid arteries (ECA). In the sham surgery group, the right ECA was ligated and 4‐0 nylon monofilament sutures (A4‐2026; Sunbio Bio.) were inserted into the ICA from the ECA. The wires were coated with poly‐L‐lysine and had rounded tips of a diameter of 5–10 mm. In pMCAO rats, the right middle cerebral artery (MCA) was occluded with a monofilament nylon thread (A4‐2026) that was coated with poly‐L‐lysine, which had a rounded tip. The wire was inserted from the ECA into the ICA until a distance of 18–20 mm was reached. The nylon thread was passed from the inside to the outside of the blood vessel. Rat body temperature was maintained using a heating pad throughout the procedure.

### 2.4 Drug administration

All treated rats were assigned into five groups: model group, sham group (Rats treated with 0.5% Na‐CMC) and piperine‐treated groups (10 and 20 mg/kg; nontoxic doses of piperine were selected based on previous research (Li et al., 2007)), and nimodipine group (treated with 12 mg/kg of nimodipine). The rats in the nimodipine group acted as the control group in this study.

### 2.5 Behavioral evaluation

There are many methods to evaluate injury severity in pMCAO model, among which the behavioral evaluation of neurological deficits is of great importance. In this study, postural reflex, body sway, balance beam, and grip strength were assessed at 3, 6, 9, 12, and 14 days postcerebral injury, aiming to evaluate sensorimotor functions.

#### 2.5.1 Postural reflex

Postural reflex testing is usually applied in evaluating sensorimotor and behavioral abilities of contralateral limbs, reflecting striatal, and cortical lesions (Bederson, Pitts, Tsuji, Nishimura, & Davis, 1986). The rats were suspended by their tails 1 m above a flat surface and were slowly lowered down. The posture of the rats was observed throughout this procedure. A behavioral score of zero was awarded if the uninjured rats extended both forelimbs toward the table, while a score of 1 was awarded if one or both forelimbs were flexed. This was followed by the lateral push test, where the rat was placed on a sheet of plastic‐coated paper and lateral pressure was applied from behind the shoulders at both right and left directions. Failure to resist the different forces equally resulted in a behavioral score of 2, while a score of 3 was given if the rats circled to the left (Alexis et al., 1996).

#### 2.5.2 Body sway

Body sway is suitable for determination of movement asymmetry. As in the postural test, the rats were lowered by their tail from 1 m above a flat surface. Symmetrical swaying to the right and left was given a score of zero, while one point was awarded for asymmetrical swaying <30°C and two points were awarded if the asymmetry was >30°C.

#### 2.5.3 Balance beam

Balance beam test is usually used in evaluating function changes including sensation, movement, and coordination after pMCAO. The rats were trained to cross a 2 cm wide, 120 cm long beam suspended at 80 cm above the floor. A 20 × 20 × 20 cm black tube was designated as the end point and was placed at the far end of the beam. The number of falls and foot slips was counted (Li et al., 2008). A score from 0 to 6 was given based on the following criteria: 0, falling upon standing on the beam; 1, ability to balance on the beam but unable to advance; 2, falling while walking along the beam; 3, ability to walk along beam with >50% foot slippage; 4, ability to walk along beam with <50% foot slippage; 5, slipping only after walking several steps; 6, ability to cross the beam without falling. The rats were first trained for 7 days prior to performing three trials.

#### 2.5.4 Grip strength test

The grip strength test utilizes the forepaw grasping reflex as an assessment of muscle strength and neuromuscular integration (Ferrara, Bejaoui, Seyen, Tirelli, & Plumier, 2009). The YLS‐12A grip strength meter (Beijing Zhongshi Dichaung Technology Development Co., Ltd.) was used to assess the grip strength of the rats. The rats were held by their tails with their forelimbs on the grid (10 × 10 cm), which was connected to a high‐precision force sensor that automatically recorded the strongest pull force. Three readings were obtained to calculate an average value, which was used for further analysis.

### 2.6 Measurement of cerebral infarct area

All rats were sacrificed via intraperitoneal injection using 10% chloral hydrate 14 days postischemic induction. Their brains were dissected, and six 2 mm sections were prepared using a rat brain matrix (Muromachi Kikai Co., Ltd.). All sections were exposed for 30 min to 2% TTC solution at 37°C. Image J software was used to evaluate the size of the infarction.

### 2.7 Histopathological analysis

All rats were sacrificed via intraperitoneal injection using 10% chloral hydrate 14 days postischemic induction. The harvested brains were rinsed in cold saline for 10 min prior to overnight fixation with 10% formalin at room temperature. 4‐ to 5‐mm‐thick coronal slices were prepared for hematoxylin and eosin staining.

### 2.8 Ultrastructural analysis

In brief, rat cerebral cortices were harvested after cerebral perfusion and the sections (1 mm^3^) were soaked in % glutaraldehyde (Tedpella) for 2 hr. The slices were postfixed using 0.1 M phosphate buffer solution with 1% osmium tetroxide for 2 hr. After being dehydrated using a series of rising ethanol solutions and epipropylene oxide, all the sections were inserted into the surface of the resin block. Ultrathin slices (70 nm) were cut and collected on Formvar‐coated copper slot grids. The samples were stained with uranyl acetate and then stained with lead citrate. The samples were observed under a transmission electron microscope (HITACHI; H‐600, JEM‐100SX).

### 2.9 Western blot analysis

All rats were sacrificed 14 days after pMCAO. The cerebral cortex and hippocampus were rapidly separated, snap‐frozen, and preserved at −80°C. A lysis buffer (Vazyme) was used to extract cortical proteins. BCA was used to determine protein quantity. Sodium dodecyl sulfate polyacrylamide gel (Bax, Bcl‐2, Caspase‐9, Caspase‐3, Cyt‐c [all 1:500; Proteintech], and β‐actin [1:1,000; CST, USA]) electrophoresis was used to separate the proteins, which were then electroblotted onto polyvinylidene difluoride (PVDF) membranes. The PVDF membranes were sealed with 5% nonfat milk at room temperature for 1 hr and subsequently incubated overnight with the primary antibodies at 4°C. This was followed by a 2 hr of incubation period with horseradish peroxidase coupling secondary antibody on the next day. Enhanced chemiluminescence was used to detect protein bands. Detection and analysis of chemiluminescence signals were achieved using a ChemiDoc XRS imaging system (Bio‐Rad).

### 2.10 Statistical analysis

The data are expressed as mean ± *SE*. One‐way ANOVA, followed by Tukey's multiple comparison test, was used to compare differences across groups. A *p* value of <.05 was interpreted as significant, while a *p* value of <.01 was interpreted as being highly significant. The data were analyzed using GraphPad Prism 6 software.

## 3 RESULTS

### 3.1 Behavioral evaluation

#### 3.1.1 Postural reflex

**Figure 1:**
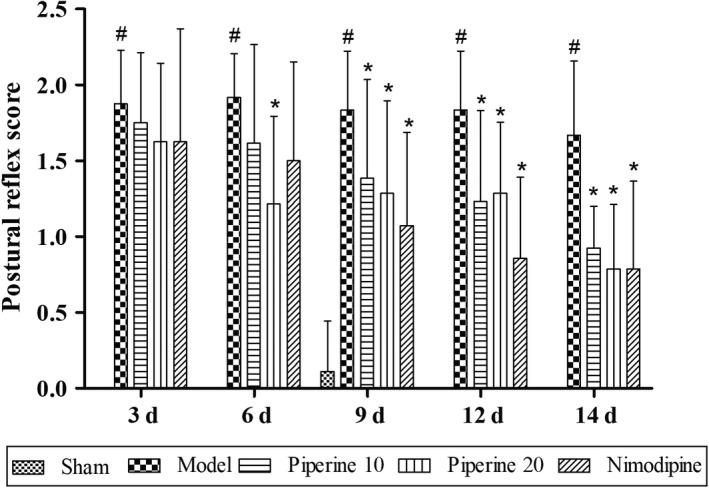
Effects of piperine on postural reflex in pMCAO model rats. Results are expressed as mean ± *SD*. ^#^ *p* < .05, versus sham group; *p < .05, versus model group (*n* = 9~13)

No obvious neurological impairment was observed in the sham group (Figure 1). The postural reflex scores were found to be markedly elevated in the model group, compared with that of the sham group (*p* < .05). However, the postural reflex scores of the rats in the piperine‐treated group (10 mg/kg) and nimodipine group were found to have decreased significantly at 9, 12, and 14 days (*p* < .05). Postural reflex scores of the piperine‐treated group (20 mg/kg) were found to have decreased significantly at 6, 9, 12, and 14 days (*p* < .05).

#### 3.1.2 Body sway

**Figure 2:**
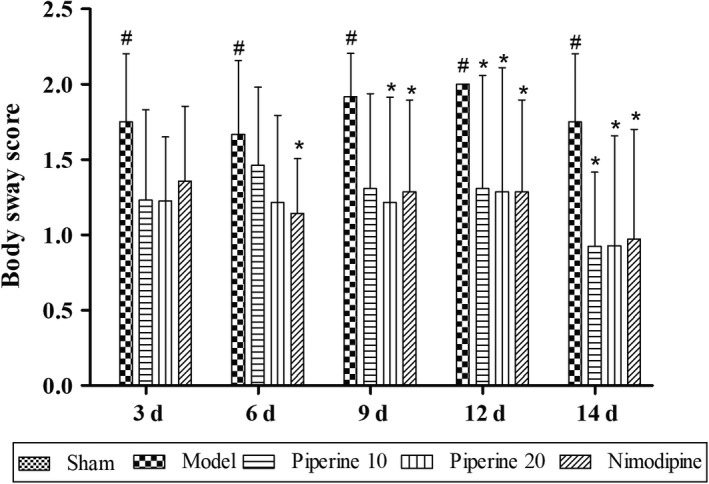
Effects of piperine on body sway in pMCAO model rats. Results are expressed as mean ± *SD*. ^#^ *p* < .05, versus sham group; *p < .05, versus model group (*n* = 9~13)

As shown in Figure 2, no obvious neurological impairment was found in the sham operation group. A statistically significant increase of the body sway score was observed in the model group, compared with that of the sham group (*p* < .05). The body sway score of the rats in the piperine‐treated group (10, 20 mg/kg) was found to have significantly decreased at 9, 12, and 14 days (*p* < .05), with a significant decreased at 6, 9, 12, and 14 days (*p* < .05) also observed in the nimodipine group, compared with the model group.

#### 3.1.3 Balance beam

**Figure 3:**
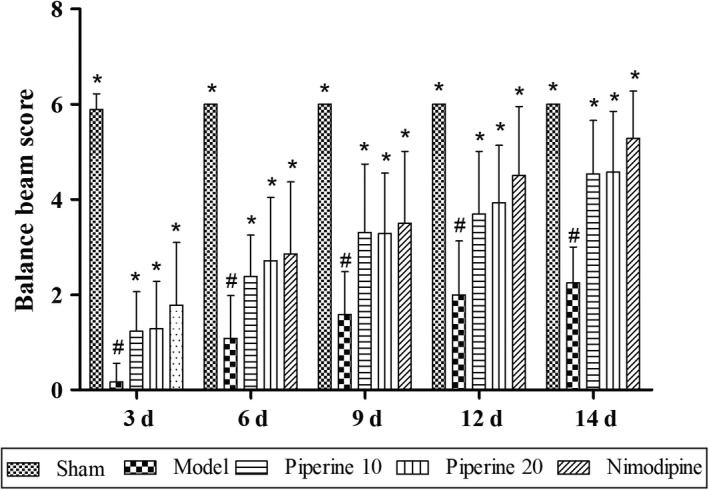
Effects of piperine on balance beam in pMCAO model rats. Results are expressed as mean ± *SD*. ^#^ *p* < .05, versus sham group; *p < .05, versus model group (*n* = 9~13)

The balance beam scores of the model group were found to have decreased significantly (*p* < .05), compared with the scores of the sham group (Figure 3). On the other hand, the balance beam scores of the rats in the piperine‐treated group and nimodipine group were found to show significant increases at 3, 6, 9, 12, and 14 days (*p* < .05), compared with the model group.

#### 3.1.4 Grip strength test

**Figure 4:**
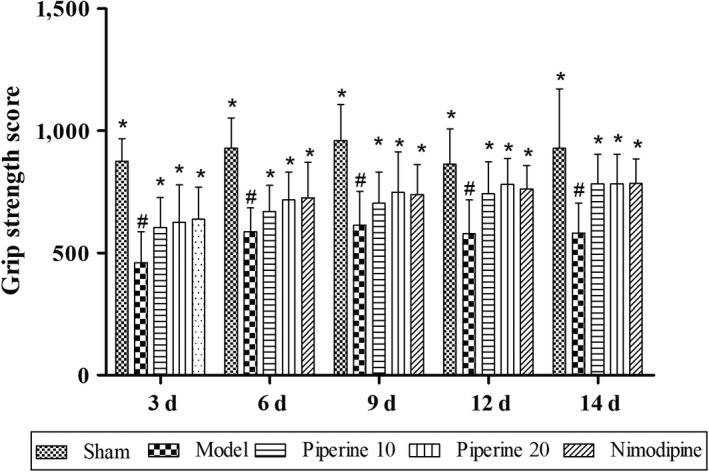
Effects of piperine on grip strength in pMCAO model rats. Results are expressed as mean ± *SD*. ^#^ *p* < .05, versus sham group; *p < .05, versus model group (*n* = 9~13)

As shown in Figure 4, the grip strength test scores of pMCAO control rats were found to have significantly reduced, compared with that of the sham rats (*p* < .05). In comparison with the model group, the grip strength test scores of the piperine‐treated group and nimodipine group were found to have significantly increased at 3, 6, 9, 12, and 14 days (*p* < .05).

### 3.2 Evaluation of cerebral infraction area

**Figure 5:**
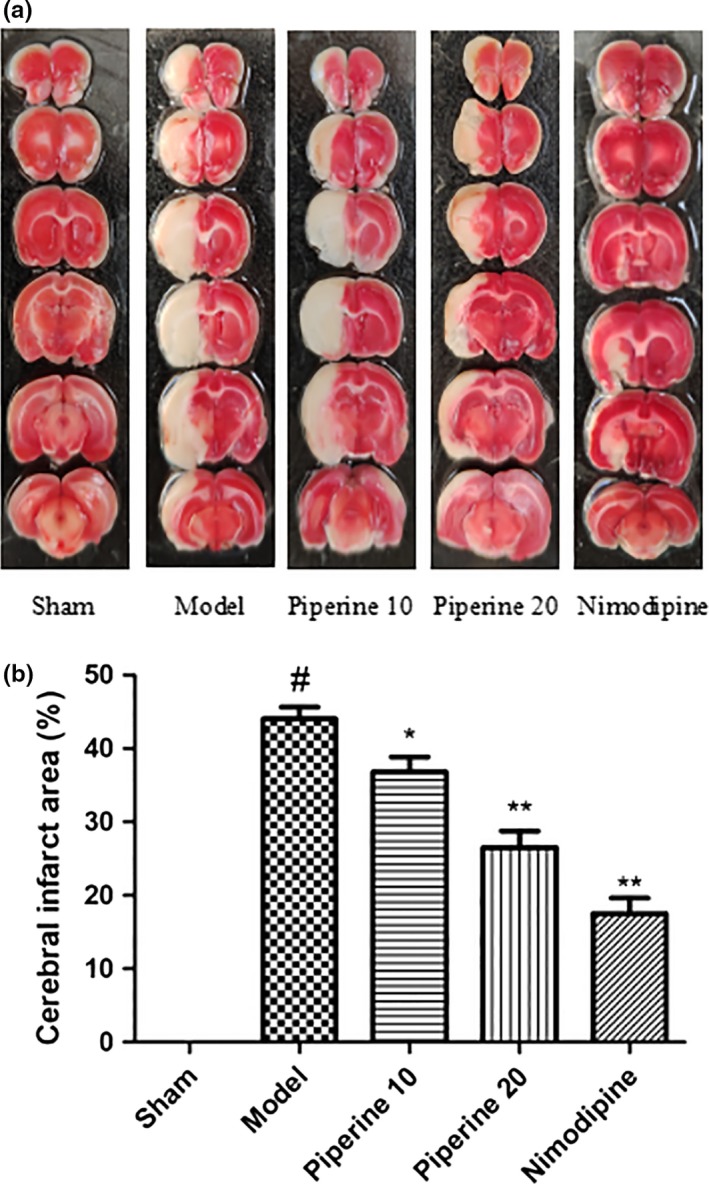
Postpiperine treatment induced changes to the ischemic area. (a) Staining with TTC on the model group, sham group, piperine treatment groups as well as the nimodipine group. (b) Measurements of the area of cerebral ischemia. **p < 0.01in contrast to the model group, ^#^ *p* < .05, in contrast to the sham group; *p < .05, (*n* = 9~13)

Cerebral samples of rats in the sham group did not show any evidence of infarction at 14 days after pMCAO. Rats in the piperine‐treated group showed different degrees of cerebral infarctions, compared with that of the model group (Figure 5a). The size of cerebral ischemia was found to have markedly reduced postpiperine treatment (*p* < .05; *p* < .01; Figure 5b).

### 3.3 Histopathological analysis

**Figure 6:**
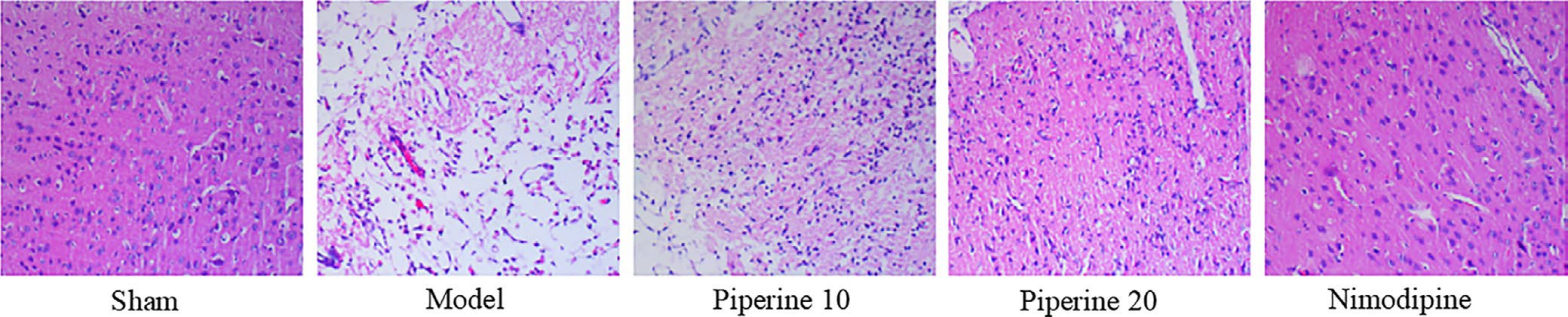
HE staining of brain samples of cerebral ischemia rats that received piperine treatment

As is shown in Figure 6, cerebral ischemia was most notable in the subcortical regions, the striatum, and in areas of the cerebral cortex. Rats of the sham group did not demonstrate histopathological evidence of cerebral injury. The brain samples of these rats demonstrated orderly neurons, normal‐sized capillaries, and glial cells with relatively intact nuclear membranes and prominent nucleoli. The ischemic brain samples were marked by neuronal edema, atrophy, triangular‐shaped nuclei, and cytoplasmic eosinophilia. The core of the ischemic regions was surrounded by injured neurons. The rats that were treated with piperine possessed smaller cerebral ischemic cores. The improvement was most marked observed in the 20 mg/kg piperine treatment group, compared with the model group.

### 3.4 Ultrastructural analysis

**Figure 7:**
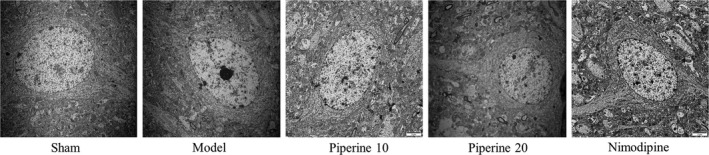
Macroscopic changes of brain samples of cerebral ischemia rats that received piperine treatment

As is shown in Figure 7, the sham operation group showed an abundance of nerve cells, oval‐shaped nucleus, no notches, even chromatin distribution, and abundant organelles. In the model group, the nuclei were irregular, with marked notches, chromatin was sparse, mitochondria were swollen, and some ridges were dissolved. In the piperine group (10 mg/kg), the nuclei were round, and chromatin was distributed evenly, but the mitochondria were swollen, sparse, and fractured. Compared with the sham operation group, the ultrastructure of nerve cells and microvessels in the piperine administration group (20 mg/kg) was normal, with mild mitochondrial swelling. In comparison with the sham group, the ultrastructure of nerve cells and microvasculature of the positive control‐treated group were normal.

### 3.5 Western blotting assessment of proteins related to apoptosis

Findings from our previous experiments suggest that piperine imparts a neuroprotective effect in post‐pMCAO‐induced brain injury rats. We hypothesized that this protective effect may be related to the expression of apoptosis‐linked proteins. In order to further explore this relationship, we quantified the expressions of Bcl‐2, Bax, Caspase‐9, Caspase‐3, and Cyt‐c in brain cortex regions using western blotting analysis.

**Figure 8:**
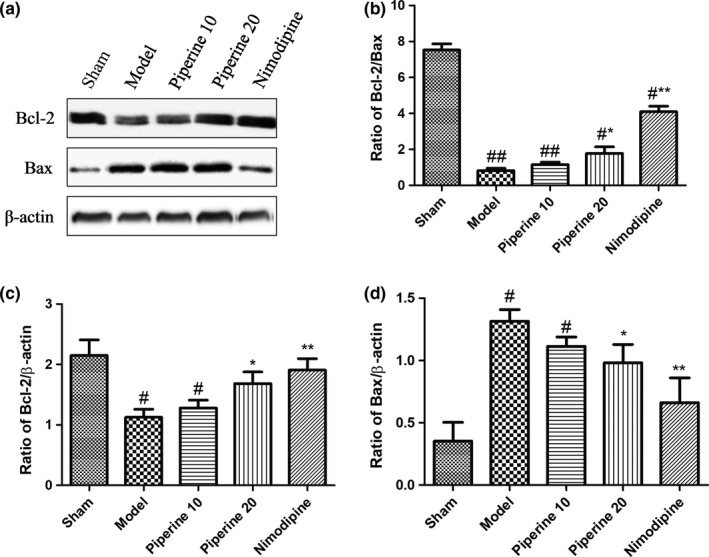
Effects of piperine on expression of Bcl‐2 and Bax in the cortex after pMCAO. (a) Represents western blot analysis of Bcl‐2 and Bax, β‐actin is shown as loading control. (b–d) Represents quantitative analysis of expression of Bcl‐2/Bax, Bcl‐2 and Bax. Data are expressed as means ± *SD*; *n* = 4. ^#^ *p* < .05, ^##^ *p* < .01, versus sham group; *p < .05, **p < .01, versus model group

As is shown in Figure 8, Bcl‐2 expression was markedly raised, while Bax expression was markedly attenuated in the model group, in contrast with that of the sham group. Rats in the piperine group (20 mg/kg) had a significantly enhanced expression of Bcl‐2 and attenuated Bax expression (*p* < .05). The Bcl‐2/Bax ratio of the model group drastically decreased (*p* < .01), whereas treatment with piperine (20 mg/kg) observably lowered (*p* < .05) the ratio in both groups, compared with that of rats in the sham group.

**Figure 9:**
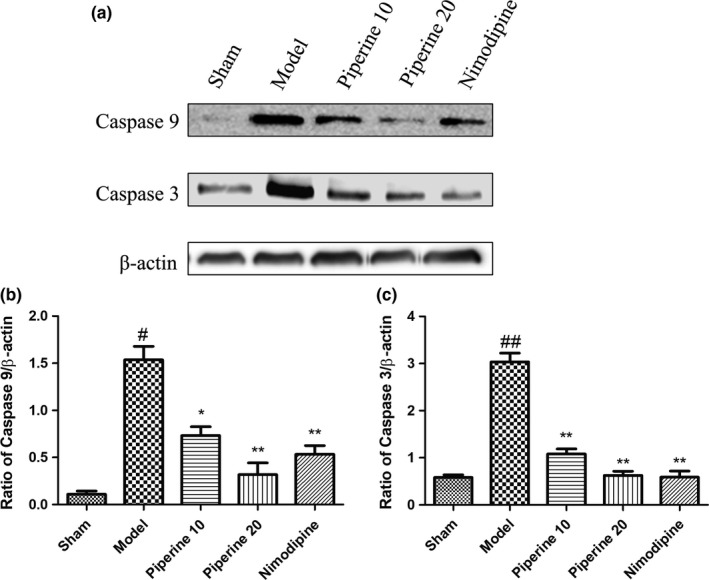
Effects of piperine on expression of Caspase‐9 and Caspase‐3 in the cortex after pMCAO. (a) Represents western blot analysis of Caspase‐9 and Caspase‐3, β‐actin is shown as loading control. (b–c) Represents quantitative analysis of expression of Caspase‐9 and Caspase‐3. Data are expressed as means ± *SD*; *n* = 4. ^#^ *p* < .05, ^##^ *p* < .01, versus sham group; *p < .05, **p < .01, versus model group

As is shown in Figure 9, compared with the sham group, the expression of the proteins, Caspase‐9 and Caspase‐3, in the model group increased significantly. Treatment with piperine (10 and 20 mg/kg) inhibited the protein expression of Caspase‐9 and Caspase‐3 (*p* < .05; *p* < .01, respectively). The groups treated with nimodipine and piperine (20 mg/kg) did not display markedly different protein profiles.

**Figure 10:**
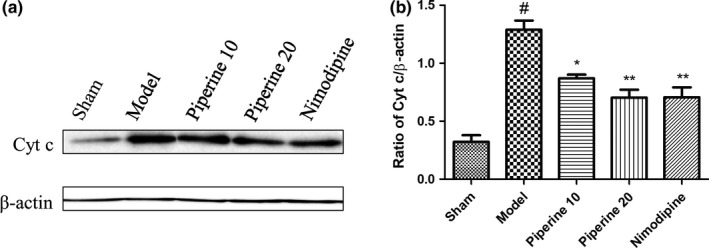
Effects of piperine on expression of Cyt‐c in the cortex after pMCAO. (a) Represents western blot analysis of Cyt‐c, β‐actin is shown as loading control. (b) Represents quantitative analysis of expression of Cyt‐c. Data are expressed as means ± *SD*; *n* = 4. ^#^ *p* < .05, ^##^ *p* < .01, versus sham group; *p < .05, **p < .01, versus model group

As is shown in Figure 10, compared with the sham group, the protein expression of Cyt‐c in the model group increased significantly. Treatment with piperine (10 and 20 mg/kg) suppressed Cyt‐c protein expression (*p* < .05; *p* < .01, respectively). No significant difference was observed between the nimodipine and piperine (20 mg/kg) groups.

## 4 DISCUSSION

Oral piperine treatment has been shown to effectively reduce the area of cerebral injury, improve behavioral impairment, and decrease the severity of cellular damage after pMCAO injury. Our results confirm that piperine can provide neuroprotection through the regulation of antiapoptotic mechanisms.

Recent studies have revealed that ischemic stroke is an important contributor to long‐term disability and morbidity worldwide (Donnan, Fisher, Macleod, & Davis, 2008). The pMCAO model has been widely established as an effective model for use in stroke research (Mcbride & Zhang, 2017), and is well‐suited for studying the pathophysiology of neuronal ischemic injury (Zou et al., 2016). Therefore, this model was used in our study to evaluate the neuroprotective effects of piperine.

Our findings demonstrate that piperine imparts a dose‐dependent neuroprotective effect, when orally administered to rat pMCAO models at doses of 10 and 20 mg/kg. pMCAO rats demonstrate significant motor dysfunction, as evidenced by an increase in turning behavior. The MCA territory houses both the pyramidal tracts and the motor cortices. Damage to this crucial location results in irreversible loss of function and inhibits the transmission of messages to the subcortical areas (Bolay & Dalkara, 1988). Piperine is able to improve several neurological deficits in pMCAO rats, with the treated rats scoring better in grip strength, beam balancing, body sway, and postural reflex test.

The histological analyses yielded similar results. TTC stains allow us to quantify the infarcted middle cerebral artery area. Using HE staining, the cells in the ischemic area were found to show swelling, nuclear atrophy, and degeneration. Microscopic neuronal changes were also observed. In conclusion, these findings objectively demonstrate the neuroprotective effects of piperine.

Apoptosis is a pivotal contributor to the pathogenesis and prognosis of several cerebrovascular diseases (Dong et al., 2014). The Bcl‐2 protein family is known to regulate programmed cell death, as well as cellular differentiation and proliferation, during the process of normal brain development (Mishra, Randis, Ashraf, & Delivoria‐Papadopoulos, 2006). In order to prevent apoptosis and protect cells from hypoxia‐induced programmed cell death, heterodimers are formed between Bcl‐2 and apoptotic protein Bax (Martinou et al., 1994). Previous studies on rats exposed to ischemic injury indicated elevated Bax levels, but decreased Bcl‐2 protein level (Wang et al., 2014). Consistent with previous research results, this study shows that oral treatment with piperine (10 and 20 mg/kg) is able to suppress Bax protein increase, while increasing Bcl‐2 protein levels, raising the Bcl‐2/Bax ratio, indicating that piperine inhibits cerebral ischemic injury by upregulating Bcl‐2 and downregulating Bax proteins.

Mitochondrial apoptosis is crucial for facilitating the pathological process of ischemic brain injury, which is triggered by Cyt‐c release from the mitochondrial intermembrane space (Broughton et al., 2009). dATP is required to facilitate Apaf‐1 and Cyt‐c binding, a key process that enhances Caspase‐9 activation, which subsequently splits and triggers the activity of executors, Caspase‐7 and Caspase‐3. Ischemic brain tissue contains increased levels of Caspase‐9 (Chen et al., 2015), and Caspase‐3 is an important mediator of apoptosis in cells subjected to ischemic stroke (Endres et al., 1998; Lu, Deng, & Lu, 2012; Ma, Endres, & Moskowitz, 2010). Our study found that piperine (10 and 20 mg/kg) was able to maintain low levels of Cyt‐c, Caspase‐3, and Caspase‐9 in permanent cerebral ischemia injury, suggesting that piperine may exert a neuroprotective effect via regulation of Cyt‐c, Caspase‐9, and Caspase‐3 proteins.

## 5 CONCLUSIONS

This study provides evidence of the efficacy of piperine in conferring neuroprotection in pMCAO rat models. Piperine has the potential to be a novel therapeutic agent in stroke treatment. However, further studies are required to explore its underlying mechanism of action.

## CONFLICT OF INTEREST

The authors declare no conflict of interest.

## ETHICAL APPROVAL

All animal care and experimental protocols were ethically reviewed and approved by the Ethics Committee of Ningxia Medical University.

## References

[fsn31185-bib-0001] Alexis, N. , Back, T. , Zhao, W. , Dietrich, W. , Watson, B. , & Ginsberg, M. (1996). Neurobehavioral consequences of induced spreading depression following photothrombotic middle cerebral artery occlusion. Brain Research, 706(2), 273–282. 8822367 10.1016/0006-8993(95)01180-3

[fsn31185-bib-0002] Bae, G. S. , Kim, M. S. , Jung, W. S. , Seo, S. W. , Yun, S. W. , Kim, S. G. , … Park, S. J. (2010). Inhibition of lipopolysaccharide‐induced inflammatory responses by piperine. European Journal of Pharmacology, 642(1), 154–162. 20621590 10.1016/j.ejphar.2010.05.026

[fsn31185-bib-0003] Bederson, J. , Pitts, L. , Tsuji, M. , Nishimura, M. , & Davis, R. (1986). Rat middle cerebral artery occlusion: Evaluation of the model and development of a neurologic examination. Stroke, 17(3), 472–476. 3715945 10.1161/01.str.17.3.472

[fsn31185-bib-0004] Bolay, H. , & Dalkara, T. (1988). Mechanisms of motor dysfunction after transient MCA occlusion: Persistent transmission failure in cortical synapses is a major determinant. Stroke, 29(9), 1988–1993. 10.1161/01.str.29.9.1988 9731628

[fsn31185-bib-0005] Broughton, B. R. S. , Reutens, D. C. , & Sobey, C. G. (2009). Apoptotic mechanisms after cerebral ischemia. Stroke, 40(5), e331. 19182083 10.1161/STROKEAHA.108.531632

[fsn31185-bib-0006] Chen, S. , Peng, H. , Rowat, A. , Feng, G. , Zhang, Z. , Peng, W. , … Qu, L. (2015). The effect of concentration and duration of normobaric oxygen in reducing caspase‐3 and ‐9 expression in a rat‐model of focal cerebral ischaemia. Brain Research, 1618, 205–211. 26032740 10.1016/j.brainres.2015.05.027

[fsn31185-bib-0007] Chu, H. X. , Kim, H. A. , Lee, S. , Broughton, B. R. , Drummond, G. R. , & Sobey, C. G. (2016). Evidence of CCR7‐independent transmigration of Ly6C(hi) monocytes into the brain after permanent cerebral ischemia in mice. Brain Research, 1637, 118–127. 26921777 10.1016/j.brainres.2016.02.030

[fsn31185-bib-0008] Costantino, I. , & Josef, A. (2012). The immunology of stroke: From mechanisms to translation. Nature Medicine, 17(7), 796–808. 10.1038/nm.2399 PMC3137275 21738161

[fsn31185-bib-0009] Dong, L. I. , Liu, M. , Tao, T. Q. , Song, D. D. , Liu, X. H. , & Shi, D. Z. (2014). Effects of Panax quinquefolium saponin on mitochondrial membrane potential and cardiomyocyte apoptosis in ischemia/reperfusion rats. Chinese Journal of Pathophysiology, 30(9), 1559–1566.

[fsn31185-bib-0010] Donnan, G. , Fisher, M. , Macleod, M. , & Davis, S. (2008). Stroke. Lancet, 371(9624), 1612–1623. 18468545 10.1016/S0140-6736(08)60694-7

[fsn31185-bib-0011] Endres, M. , Namura, S. , Shimizu‐Sasamata, M. , Waeber, C. , Zhang, L. , Gómez‐Isla, T. , … Moskowitz, M. A. (1998). Attenuation of delayed neuronal death after mild focal ischemia in mice by inhibition of the caspase family. Journal of Cerebral Blood Flow and Metabolism, 18(3), 238–247. 9498840 10.1097/00004647-199803000-00002

[fsn31185-bib-0012] Ferrara, A. , Bejaoui, S. E. , Seyen, S. , Tirelli, E. , & Plumier, J. C. (2009). The usefulness of operant conditioning procedures to assess long‐lasting deficits following transient focal ischemia in mice. Behavioural Brain Research, 205(2), 525–534. 19686784 10.1016/j.bbr.2009.08.011

[fsn31185-bib-0013] GBD 2016 Causes of Death Collaborators (2017). Global, regional, and national age‐sex specific mortality for 264 causes of death, 1980–2016: A systematic analysis for the global burden of disease study 2016.%A. Lancet (London, England), 390(10100), 1151–1210. 10.1016/S0140-6736(17)32152-9 PMC5605883 28919116

[fsn31185-bib-0014] GBD 2016 DALYs and HALE Collaborators (2017). Global, regional, and national disability‐adjusted life‐years (DALYs) for 333 diseases and injuries and healthy life expectancy (HALE) for 195 countries and territories, 1990–2016: A systematic analysis for the global burden of disease study 2016. Lancet, 390(10100), 1260–1344. 28919118 10.1016/S0140-6736(17)32130-X PMC5605707

[fsn31185-bib-0015] Graham, S. H. , & Chen, J. (2001). Programmed cell death in cerebral ischemia. Journal of Cerebral Blood Flow and Metabolism, 21(2), 99–109. 11176275 10.1097/00004647-200102000-00001

[fsn31185-bib-0016] Hyo‐Soon, J. , Hye‐Yeon, C. , Tae‐Won, C. , Bong‐Woo, K. , Jung‐Hyun, K. , Eung‐Ryoung, L. , & Ssang‐Goo, C. (2008). Differential regulation of the antiapoptotic action of B‐cell lymphoma 2 (Bcl‐2) and B‐cell lymphoma extra long (Bcl‐xL) by c‐Jun N‐terminal protein kinase (JNK) 1‐involved pathway in neuroglioma cells. Biological & Pharmaceutical Bulletin, 31(9), 1686. 18758060 10.1248/bpb.31.1686

[fsn31185-bib-0017] Li, L. , Li, Y. , Ji, X. , Zhang, B. , Wei, H. , & Luo, Y. (2008). The effects of retinoic acid on the expression of neurogranin after experimental cerebral ischemia. Brain Research, 1226, 234–240. 18602376 10.1016/j.brainres.2008.06.037

[fsn31185-bib-0018] Li, S. , Wang, C. , Wang, M. , Li, W. , Matsumoto, K. , & Tang, Y. (2007). Antidepressant like effects of piperine in chronic mild stress treated mice and its possible mechanisms. Life Sciences, 80(15), 1373–1381. 17289085 10.1016/j.lfs.2006.12.027

[fsn31185-bib-0019] Liu, G. Q. , Algeri, S. , Ceci, A. , Garattini, S. , Gobbi, M. , & Murai, S. (1986). Stimulation of serotonin synthesis in rat brain after antiepilepsirine, an antiepileptic piperine derivative. Chinese Medical Journal, 33(23), 3883–3886. 10.1016/0006-2952(84)90055-8 6210090

[fsn31185-bib-0020] Lu, Z. Q. , Deng, Y. J. , & Lu, J. X. (2012). Effect of aloe polysaccharide on caspase‐3 expression following cerebral ischemia and reperfusion injury in rats. Molecular Medicine Reports, 6(2), 371–374. 22641427 10.3892/mmr.2012.927

[fsn31185-bib-0021] Luo, Y. , Yang, Y. P. , Liu, J. , Li, W. H. , Yang, J. , Sui, X. , … Chen, D. (2014). Neuroprotective effects of madecassoside against focal cerebral ischemia reperfusion injury in rats. Brain Research, 1565(20), 37–47. 24735651 10.1016/j.brainres.2014.04.008

[fsn31185-bib-0022] Ma, J. , Endres, M. , & Moskowitz, M. A. (2010). Synergistic effects of caspase inhibitors and MK‐801 in brain injury after transient focal cerebral ischaemia in mice. British Journal of Pharmacology, 124(4), 756–762. 10.1038/sj.bjp.0701871 PMC1565432 9690868

[fsn31185-bib-0023] Mao, Q. Q. , Huang, Z. , Zhong, X. M. , Xian, Y. F. , & Ip, S. P. (2014). Piperine reverses the effects of corticosterone on behavior and hippocampal BDNF expression in mice. Neurochemistry International, 74(13), 36–41. 24816193 10.1016/j.neuint.2014.04.017

[fsn31185-bib-0024] Martinou, J. C. , Dubois‐Dauphin, M. , Staple, J. K. , Rodriguez, I. , Frankowski, H. , Missotten, M. , … Pietra, C. (1994). Overexpression of BCL‐2 in transgenic mice protects neurons from naturally occurring cell death and experimental ischemia. Neuron, 13(4), 1017. 7946326 10.1016/0896-6273(94)90266-6

[fsn31185-bib-0025] Mcbride, D. W. , & Zhang, J. H. (2017). Precision stroke animal models: The permanent MCAO model should be the primary model, not transient MCAO. Translational Stroke Research, 8(12), 1–8. 28718030 10.1007/s12975-017-0554-2 PMC5772000

[fsn31185-bib-0026] Mishra, A. , Punia, J. K. , Bladen, C. , Zamponi, G. W. , & Goel, R. K. (2015). Anticonvulsant mechanisms of piperine, a piperidine alkaloid. Channels, 9(5), 317–323. 26542628 10.1080/19336950.2015.1092836 PMC4826125

[fsn31185-bib-0027] Mishra, O. P. , Randis, T. , Ashraf, Q. M. , & Delivoria‐Papadopoulos, M. (2006). Hypoxia‐induced Bax and Bcl‐2 protein expression, caspase‐9 activation, DNA fragmentation, and lipid peroxidation in mitochondria of the cerebral cortex of newborn piglets: The role of nitric oxide. Neuroscience, 141(3), 1339–1349. 16777344 10.1016/j.neuroscience.2006.05.005

[fsn31185-bib-0028] Rameshkumar, K. B. , Anuaravind, A. P. , & Mathew, P. J. (2011). Comparative phytochemical evaluation and antioxidant assay of *Piper longum* L. and Piper chaba hunter used in Indian traditional systems of medicine. Journal of Herbs Spices & Medicinal Plants, 17(4), 351–360.

[fsn31185-bib-0029] Reed, J. C. (2000). Mechanisms of apoptosis. American Journal of Pathology, 157(5), 1415–1430. 11073801 10.1016/S0002-9440(10)64779-7 PMC1885741

[fsn31185-bib-0030] Shi, S. S. , Yang, W. Z. , Chen, Y. , Chen, J. P. , & Tu, X. K. (2014). Propofol reduces inflammatory reaction and ischemic brain damage in cerebral ischemia in rats. Neurochemical Research, 39(5), 793–799. 24610527 10.1007/s11064-014-1272-8

[fsn31185-bib-0031] Wang, G. H. , Lan, R. , Zhen, X. D. , Zhang, W. , Xiang, J. , & Cai, D. F. (2014). An‐Gong‐Niu‐Huang Wan protects against cerebral ischemia induced apoptosis in rats: Up‐regulation of Bcl‐2 and down‐regulation of Bax and caspase‐3. Journal of Ethnopharmacology, 154(1), 156–162. 24690773 10.1016/j.jep.2014.03.057

[fsn31185-bib-0032] Zhang, H. , Zhou, X. , Wong, M. H. I. , Man, K. I. , Pin, W. W. , Yeung, J. H. E. , … Lee, S. M. U. (2017). Sichuan pepper attenuates H2O2‐induced apoptosis via antioxidant activity and up‐regulating heme oxygenasegene expression in primary rat hepatocytes. Journal of Food Biochemistry, 41, e12403.

[fsn31185-bib-0033] Zhang, L. , Lam, W. P. , Lü, L. , Wang, C. , Wong, Y. W. , Lam, L. H. , … Kwong, W. H. (2010). Protective effects and potential mechanisms of Pien Tze Huang on cerebral chronic ischemia and hypertensive stroke. Chinese Medicine, 5(1), 35. 20955558 10.1186/1749-8546-5-35 PMC2984508

[fsn31185-bib-0034] Zou, H. , Long, J. , Zhang, Q. , Zhao, H. , Bian, B. , Wang, Y. , … Lei, W. (2016). Induced cortical neurogenesis after focal cerebral ischemia–Three active components from Huang‐Lian‐Jie‐Du Decoction. Journal of Ethnopharmacology, 178, 115–124. 26657578 10.1016/j.jep.2015.12.001

